# 

**Published:** 1881-07

**Authors:** 


PAGE

ORIGINAL COMMUNICATIONS—The Effect of Muscular Exertion on Proteid Metabolism—The Standard Rations for Working Animals. By H. P. Ormsby, Ph.D. . . . . . . . . . . . . . . . 151
The Cause and Cure of Disease in Horses' Feet. By E. A. McLellan . . . . . . . . . . . . . 162
On the Lesions and Pathology of the So-called Posterior Paralysis of the Horse. By William Henry Porter, M.D., D.V.S., and George H. Parkinson, D.V.S. . . . . . . . . . . . . . . . . 170

EDITORIAL—Shall Our Horses Wear Shoes?—The New Commissioner of Agriculture—Doctoring Through the Newspapers—Preventing Diseases by Inoculation—The Veterinary Medical Register—Breeding in its Relations to Physiology and Pathology—Phagedæna—Is it a New Disease? . 178–186

CASE DEPARTMENT—Lacerated Wound of Hip—Lacerated Wound of Tongue: Amputation—Removal of Fatty Fibroma—Acute Congestion of the Lumbar Region of the Spinal Cord, Cystitis, Pyelitis, Acute Parenchymatous Nephritis—Posterior Paralysis of the Horse—Notes on Autopsies made at Columbia Veterinary College and School of Comparative Medicine—Concretions in Large Intestine of a Swan—Bloody Infiltration of Pelvic Cellular Tissue in a Doe—Extensive Caseous Deposits in the Lungs of a Drill . . . . . . . . . . . . . . . . . . . . . . . . . . . . 187–192

CORRESPONDENCE—Singular Instance of Depraved Appetite in a Monkey—Preventing Diseases by Inoculation—Uræmic Convulsions in a Pug—Curious Veterinary Experience—The Physiological Aspects of Breeding—Phagedæna, a New Disease? . . . . . . . . . . . . . . . . . . . . . 193–201

PROGRESS OF VETERINARY SCIENCE—Vegetable Charcoal—Gout in Parrots—Splenic Leucocythemia—Fibrinous Endocarditis—Rabies in the Horse—Iodoform—Red Blood Globules—Anthrax Poison in Soils—A New Cestoid Parasite—Trichinæ in Chickens—A Canine Epileptic Affection caused by Acari—Persistent Effect of Animal Poisons—Effect of Lightning upon Animals—Trance in the Lower Animals—Diseases of Animals in Captivity—Farcy—Dropsy in Horses—Rupture of Horse's Stomach—Laws Relating to Contagious Diseases of Animals in Germany—Round Worms in the Alimentary Tract of Sheep . . . . . . . . . . . . . . . . . . . . . . . . 202–208

NEWS AND MISCELLANY—New Veterinary School—Flaxseed Meal—Cross-eyed Mule—Another Fertile Mule—New Veterinary Publication—Bi-chromate of Potassa—New Cause for Pleuro-Pneumonia—Fluke Parasite—Foot-and-Mouth Disease—Anthrax in the West—Horses of New York—Equina *vs.* Phagedæna—Horse in Spectacles—Pleuro-Pneumonia in England—Veterinary Congresses—Dysentery in Dogs—Deaths—Veterinary Surgeon's Bill—Veterinary Law in Illinois—American Pork—Cribbing—Destruction of Lice—Diarrhœa in Calves—Monstrosity in Fowls—Museums for Private Veterinary Practitioners—Sows Eating Pigs—Epidemic Among Deer—Bill to Prevent the Spread of Contagious Dieasses Among Animals Criticised—Dog Home and Hospital—Epidemic Among Horses in Chicago—Healthfulness of American Swine—Inversion of the Vagina of a Cow—Elephant Dentistry at the Zoo—Case of Superfetation in the Horse . . . . . . . . 2 9–214
VETERINARY MEDICAL REGISTER . . . . . . . . . . . . . . . . . . . . . . . . . . . . 215–225

